# A nationally representative survey of the impact of discrimination towards people with mental health problems: SANE’s 2025 National Stigma Report Card

**DOI:** 10.1017/S2045796026100456

**Published:** 2026-02-03

**Authors:** Nicola J Reavley, Anna M Ross, Gayle McNaught, Rachel Green, Amy J Morgan

**Affiliations:** 1 Centre for Mental Health and Community Wellbeing, Melbourne School of Population and Global Health, The University of Melbourne, Melbourne, Australia; 2 SANE, Melbourne, Australia

**Keywords:** discrimination, mental illness stigma, population survey, social inclusion, stereotypes

## Abstract

**Aims:**

Reducing stigma and discrimination has been a priority in many national mental health policies for decades. Focusing efforts requires us to understand where this has the greatest impact on people with mental health problems. In 2024, we conducted a nationally representative survey that aimed to assess the burden of discrimination (as a product of frequency and impacts of experiences). Secondary aims were to quantify the types of discrimination experienced in different life domains and the sociodemographic and mental health problem characteristics of those experiencing higher burden.

**Methods:**

Online surveys were completed by 6032 members of the general Australian community aged 18 years and over. The survey was carried out by the survey company The Social Research Centre, using their Life in Australia™ probability-based panel. Those who reported a mental health problem or scored high on the Kessler 6 measure of psychological distress (*n* = 2613) were asked about the past 12-month frequency and impact of their experiences of discrimination in a broad range of settings, including family, friends, workplaces and health services. The data were initially analysed using percent frequencies and 95% confidence intervals. A burden score was calculated for each domain, incorporating frequency and impact among those who reported discrimination experiences.

**Results:**

Overall, discrimination in social life was the most common (43.6% [95% CI 41.2, 45.9]), followed by discrimination from family (41.4% [95% CI 39.1, 43.7]) or in making or keeping friends (41.0% [95% CI 38.7, 43.3]). However, the highest burden was from discrimination in finding or keeping a job, in dating/intimate relationships, in housing (including renting or public housing) and in obtaining welfare benefits or disability pensions. The most common type of discrimination experience in the workplace and among friends, family and partners was of people lacking understanding of the impact of the person’s mental health problem. People aged 35–64 years were more likely than those aged 18–34 years to report higher burden in multiple domains; people with depression or attention-deficit/hyperactivity disorder also reported burden in more domains than people with anxiety or severe mental health conditions. Overall, 67.7% (95% CI 65.5, 69.9) agreed that stigma and discrimination was worse than the mental health problem itself.

**Conclusions:**

Our study suggests that reducing the frequency and impact of discrimination in workplaces, welfare benefits and housing should be key targets for policy and practice. Improving the capacity of people in workplaces and intimate partners, families and friendship groups to better understand the impacts of mental health problems on individuals should also be a priority.

## Introduction

Stigma is a social process that shames, rejects and devalues groups of people based on certain characteristics (Thornicroft *et al.*, 2022). Commonly held stigmatizing attitudes towards people with mental health problems include beliefs that they are weak, dangerous, incompetent and can never recover (Reavley and Jorm, 2011). Such attitudes are referred to as personal or public stigma (Thornicroft *et al.*, 2022), and they may lead to experiences of discrimination, which may involve individual behaviours, or may be structural, in which institutions and cultural norms perpetuate disadvantage (Hatzenbuehler, 2016). As a result, people with mental health problems may be reluctant to seek treatment, may experience social exclusion and be denied employment or educational opportunities (Patel *et al.*, 2018). They may have their human rights violated and experience severe poverty, particularly in low- and middle-income countries (Chisholm *et al.*, 2016).

With relatively few exceptions, most population-level stigma research has involved the exploration of attitudes towards people with mental health problems, rather than actual experiences of discrimination against people with mental health problems. Those that have recruited participants from the general population have found this to be widespread. They include a 2010 Canadian study that asked 752 respondents who had received mental health treatment in the previous year about unfair treatment or others’ negative opinions (Stuart *et al.*, 2014). Overall, 37.4% of respondents had experienced discrimination in at least one life domain, most commonly in relation to intimate personal relationships, such as family or romantic relationships. Similar findings were seen in a 2014 Australian survey of 1381 people who reported a mental health problem or scored highly on a symptom screening questionnaire (Reavley and Jorm, 2015). Questions covered experiences of avoidance, discrimination and positive treatment by friends, spouse, other family, workplace, educational institution and others in the community. Discrimination was most common in relation to friends and family (Morgan *et al.*, 2017). As part of the 2014 California Well-Being Survey, people who reported experiencing a mental health problem in the previous 12 months were asked about their experiences of discrimination (Wong *et al.*, 2017). Nearly 90% reported an experience of discrimination in the previous year with respondents experiencing this in an average of six life domains, with social relationships being the most frequently endorsed.

Several studies have explored experiences of discrimination in people recruited through mental health services, including large multi-country studies of people with diagnoses of either major depressive disorder (Lasalvia *et al.*, 2013), schizophrenia (Thornicroft *et al.*, 2009) or anxiety disorders (Lasalvia *et al.*, 2021). In these studies, being shunned or avoided by other people was the most common experience, and the domains in which discrimination was most common were in making or keeping friends and intimate relationships. With some exceptions, discrimination experiences are generally higher in people with schizophrenia (Thornicroft *et al.*, 2009; Lasalvia *et al.*, 2021).

Studies such as these point to the importance of tackling stigma and discrimination, and this is a priority in global mental health research and policy (Thornicroft *et al.*, 2022). The World Health Organisation has produced a toolkit of initiatives to tackle stigma and discrimination in mental health (World Health Organisation, 2024). In Australia, the National Mental Health Commission has drafted a nationally coordinated stigma and discrimination reduction strategy (National Mental Health Commission, 2023). Policy recommendations for tackling broad societal issues like discrimination against people with mental health problems are often very broad-ranging and can present challenges to policymakers as they manage competing demands for action and resources. Previous research has largely focused on assessing whether discrimination has occurred in key life domains but, with few exceptions (e.g., Brouwers *et al.*, 2016), has not explored the frequency or impact of those experiences. A deeper exploration of the burden of discrimination, which assists in quantifying population-level cumulative psychosocial load or practical exclusion, may assist with prioritization of efforts, including where to focus efforts to reduce structural discrimination.

Therefore, the aim of the 2025 National Stigma Report Card was to carry out a population survey of experiences of discrimination as well as the burden of these, defined as a product of the frequency and impact of these experiences. Secondary aims were to quantify the different types of discrimination experienced in different life domains, as well as the sociodemographic and mental health problem characteristics of those experiencing higher levels of discrimination.

## Methods

Online surveys were completed by 6032 members of the general community aged 18 years and over. The survey was carried out by the survey company The Social Research Centre, using their Life in Australia™ probability-based panel. A stratified random sample was drawn from panellists on strata defined by age (18–34, 35–44, 45–54, 55–64, and 65+), gender, education and speaking a language other than English at home. Panel members were sent an initial survey invitation via email and SMS (where available), followed by up to five reminders within the fieldwork period. All panel members were offered a reimbursement of $10 or $20, depending on survey length (either in the form of a gift card or a donation to a charity).

Data collection was completed in November and December 2024. Ethics approval was obtained from the University of Melbourne Human Research Ethics Committee (ID30886).

## Survey questionnaire

In this study, we report on a sub-section of the questions included in the larger 2025 National Stigma Report Card Survey. This also includes stigmatizing attitudes, self-stigma, carer stigma and exposure to media reporting of people with mental illness. These findings are reported elsewhere (e.g., Ross *et al.*, 2025). All questions were reviewed by SANE’s Lived Experience Advisory Group, and their suggested changes were incorporated.

The following sociodemographic information was collected: state, capital city/rest of state, age, gender, citizenship status, Aboriginal and Torres Strait Islander status, country of birth, highest level of primary/secondary school completed, level of the highest educational qualification, employment status, marital status and family composition.

### Mental health and psychological distress

Respondents were taken through the 12-month version of the Kessler 6 (K6) psychological distress screening questionnaire (Kessler *et al.*, 2010), which involves asking participants to think about one month in the last 12 months when they were most depressed, anxious or emotionally stressed. Response options were 1 = None of the time, 2 = A little of the time, 3 = Some of the time, 4 = Most of the time, and 5 = All of the time. Respondents were also asked whether, over the last 12 months, they had experienced any sort of mental health problem (defined in the preamble to the question in the following way: ‘a period of weeks or more when you are feeling depressed, anxious or emotionally stressed, and these problems are interfering with your life. Mental health problems could include, for example, depression, anxiety disorders, eating disorders, schizophrenia, bipolar disorder or personality disorders’). Those respondents who answered yes to this question were then asked what they thought the problem was. Only those respondents who specified any of the following mental health problems were considered in scope: depression/major depression, attempted suicide or self-harm, anxiety/anxiety disorder, post-traumatic stress disorder (PTSD), agoraphobia, panic disorder, obsessive-compulsive disorder (OCD), social phobia, generalized anxiety disorder (GAD), eating disorder/anorexia/bulimia, schizophrenia/paranoid schizophrenia, schizoaffective disorder, psychosis/psychotic, bipolar/bipolar disorder/manic-depressive disorder, mental illness, personality disorder/borderline personality disorder, attention-deficit/hyperactivity disorder (ADHD), Autism/Asperger’s and nervous breakdown.

At this point, survey respondents were divided into two groups: (1) those who scored in the ‘high’ range on the K6 (equal to or above 19) or who reported having had an in-scope mental health problem; and (2) those who did not meet these criteria. Further questions asked whether the person had been given a diagnosis by a health professional and what this was, whether they had received treatment and what this was.

### Experiences of discrimination

Those in the first group were then asked about times in the last 12 months when they had been treated unfairly because of the mental health problems they had experienced, in the following domains (which were presented in random order): In making or keeping friends; In your social life; In dating or by your partner/husband/wife; In starting a family or having children; By your family; By the people in your neighbourhood; In finding a job; In keeping a job; In your education or further training; By a health professional when getting help for a mental health problem; By a health professional when getting help for physical health problems; In housing (including renting and accessing public or community housing); In applying for and getting welfare benefits or disability pensions; By the legal system and On social media. These domains drew on previous surveys in Australia and other countries (e.g., Thornicroft *et al.*, 2009; Reavley and Jorm, 2015). Response options were: Not applicable, Not at all, A little, Moderately and A lot.

For each circumstance in which a person reported experiencing some unfair treatment, they were asked what impact this had, with response options of: A large negative impact, A small negative impact, No impact, A small positive impact and A large positive impact. If a respondent reported unfair treatment in a particular domain, they were then presented with a list of experiences (which varied by domain and also drew on previous surveys (Reavley and Jorm, 2015; Behavioural Economics Team of the Australian Government, 2022)) and asked if they had experienced these (with a request to select all that applied).

A further question asked respondents whether they thought that stigma and discrimination was worse than the mental health problem itself. Response options were Strongly agree, Somewhat agree, Neither, Somewhat disagree, and Strongly disagree.

### Weighting

Weighting procedures involved computation of a base weight and subsequent adjustment based on population benchmarks for specific demographic characteristics. The base weight for each respondent was the product of three weights: (1) Enrolment weight, accounting for the initial chances of selection into the panel and subsequent post-stratification to key demographic benchmarks; (2) An adjustment for probability of selection into the sample of the survey and (3) Their response propensity weight, estimated from enrolment information available for both respondents and nonrespondents to the survey. The base weight was adjusted to satisfy population benchmarks for the following: age, gender, language other than English spoken at home, highest level of education, state or territory, geographic location (all based on the 2021 Census, supplemented by the latest demographic statistics; Australian Bureau of Statistics, 2023) and the number of adults in the household (based on the 2020–21 National Health Survey; Australian Bureau of Statistics, 2022).

### Statistical analysis

The data were analysed using percent frequencies and 95% confidence intervals. To create a ‘discrimination burden score’ for each domain, frequency of experiences of discrimination (scored 1 = a little, 2 = moderately and 3 = a lot respectively) was multiplied by the severity of impact (with the response options scored as follows: −2 = large positive impact, −1 = small positive impact, 0 = no impact, 1 = small negative impact and 2 = large negative impact. Scores ranged from −6 to +6. Higher scores indicated a higher burden.

Multiple linear regression analyses were used to explore the sociodemographic characteristics (age, gender, level of education, country of birth (English speaking vs other), Aboriginal or Torres Strait Islander status and mental health problem factors associated with burden scores in each domain. Mental health problem characteristics were coded as present vs absent as follows: any anxiety problem (any of anxiety/anxiety disorder, PTSD, GAD, agoraphobia, panic disorder, social phobia/anxiety disorder and obsessive compulsive disorder), any severe mental illness (any of schizophrenia, bipolar disorder, schizoaffective disorder, psychosis and personality disorder), depression/major depression and ADHD.

The regression analyses were specified a priori to estimate adjusted associations rather than to derive parsimonious predictive models, and therefore did not employ stepwise selection procedures. The sample size was large relative to the number of predictors included, supporting adequate statistical power for estimation. Multicollinearity was assessed and found to be within acceptable limits. While multiple domain-specific outcomes were examined, findings were interpreted cautiously, with emphasis placed on consistency of associations across domains and on effect sizes and confidence intervals rather than isolated p-values (Babyak, 2004).

All analyses were performed using StataSE 18 (Stata Corp 2023, LP, Texas, USA).

## Results

In total, 6032 questionnaires were completed. The completion rate, defined as the proportion of all Life in Australia™ adult members invited to participate in the survey, was 63.7%.

Among these, 27.0% (95% CI 25.6, 28.4) respondents were found to have K6 scores of 19 or above and 39.0% (95% CI 37.5, 40.5) respondents reported having an in-scope mental health problem (see Table 1). Only 5.5% (95% CI 4.8, 6.3) reported K6 scores of 19 or above but did not report an in-scope diagnosis. The most common self-reported mental health problems were anxiety/anxiety disorders, reported by 69.5% (95% CI 67.2, 71.7) and depression/major depression, reported by 64.6% (95% CI 62.2, 66.9). Among these respondents, 67.7% (95% CI 65.4, 70.0) had received a diagnosis, and 59.7% (95% CI 57.2, 62.1) had received treatment. Thus, 44.4% (95% CI 42.9, 46.0) of the total number of respondents were asked the questions about personal experiences of discrimination. Overall, 63.6% (95% CI 62.1, 65.0) of participants were in paid employment, and 36.4% (95% CI 35.0, 37.9) were not.

**Table 1.** K6 scores and mental health problems in the last 12 months

K6/mental health problems (*n* = 6032)	*n*	Weighted % (95% CI)
**K6**		
K6 score ≥ 19	1477	27.0 (25.6, 28.4)
K6 score ≤ 18	4555	73.0 (71.6, 74.4)
**In-scope mental health problem in the last 12 months (*n* = 6032)**		
Yes	2335	39.0 (37.5, 40.5)
No	3697	61.0 (59.5, 62.5)
**Diagnosis or treatment received (*n* = 2335)**		
Received diagnosis	1574	63.9 (61.6, 66.2)
Received treatment	1437	56.8 (54.5, 59.2)
**Self-reported mental health problem (nominated by >5% of those with an in-scope mental health problem) (*n* = 2335)^a^**		
Anxiety/anxiety disorder	1594	69.5 (67.2, 71.7)
Depression	1471	64.6 (62.2, 66.9)
PTSD	534	21.5 (19.5, 23.5)
Social phobia	469	21.0 (19.1, 23.1)
GAD	454	20.3 (18.4, 22.5
ADHD	421	19.9 (17.9, 22.1)
Eating disorder	239	10.6 (9.1, 12.3)
Mental illness	206	10.6 (9.1, 12.3)
OCD	192	9.2 (7.9, 10.9)
Autism spectrum disorder	184	9.3 (7.8, 11.0)
Panic disorder	182	8.1 (6.8, 9.5)
Alcohol problem	170	7.6 (6.4, 9.1)
Nervous breakdown	152	8.0 (6.6, 9.5)
Any anxiety/anxiety problem	1943	83.4 (81.4, 85.2)
Any severe mental illness	169	8.3 (7.0, 9.9)
Scored ≥19 on K6 OR said yes to having an in-scope mental health problem (total)	2613	44.4 (42.9, 46.0)

a Note multiple categories possible.

### Experiences of discrimination

**Figure 1.**
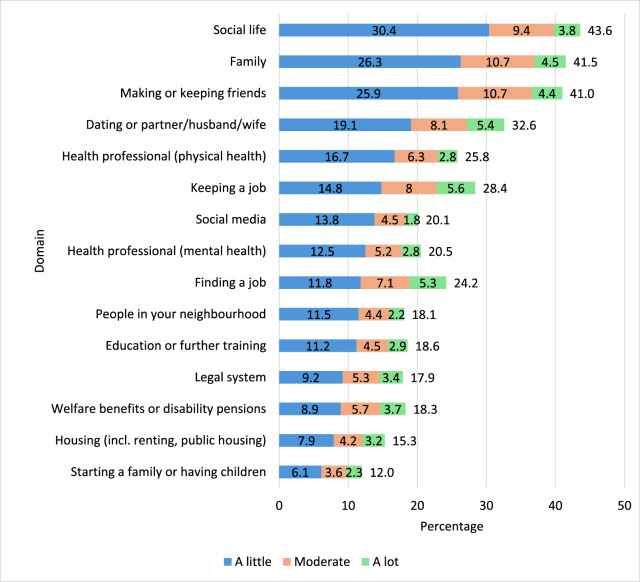
Domains and frequency of experiences of discrimination (*n* = 2613).

**Figure 2.**
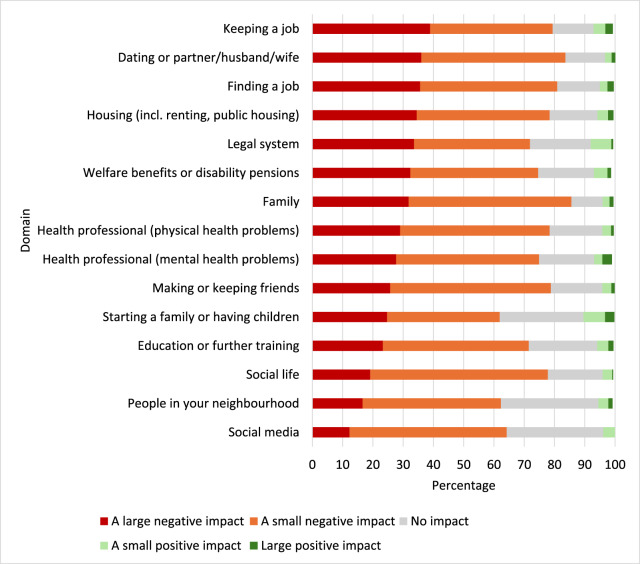
Impact of experiences discrimination.

Figure 1 gives the weighted prevalence proportions of the overall study population who reported experiences of discrimination in key domains, as well as the frequency of experiences. Overall, discrimination in social life was the most common (43.6% [95% CI 41.2, 45.9]), followed by discrimination from family (41.4% [95% CI 39.1, 43.7]) or in making or keeping friends (41.0% [95% CI 38.7, 43.3]). Discrimination in welfare benefits, in housing or in starting a family or having children were least common. However, when ranked by discrimination that occurred a lot, discrimination in keeping a job, in dating or from a partner and in finding a job were the domains in which people reported ‘a lot’ of discrimination. Figure 2 outlines the weighted prevalence proportions of reports of the impact of the experiences of discrimination, ranked by proportion of those reporting a large negative impact. Discrimination in keeping a job, in dating or from a partner and in finding a job were the domains in which people were most likely to report a large negative impact. Discrimination in social life, by people in the neighbourhood and on social media, had the least negative impact.

*N*s ranged from *n* = 347 to *n* = 1101 and only included those who reported any experience of discrimination in a particular domain

**Figure 3.**
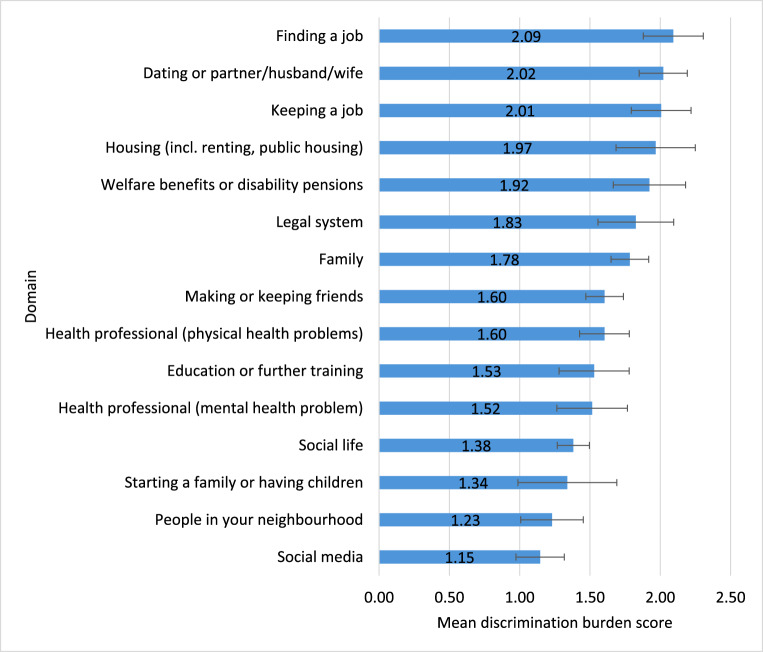
Mean scores (with 95% CIs) of burden of discrimination in different domains.

Figure 3 gives the mean scores of the burden of discrimination in different domains, as a product of the frequency and severity of experiences. Discrimination in finding a job, in dating/partners, in keeping a job, in housing (including renting or public housing) and in the area of welfare benefits or disability pensions had the highest burden for respondents.

In response to the question about whether the stigma and discrimination was worse than the mental health problem itself, responses were: Strongly agree 22.6% (95% CI 20.7, 24.5), Somewhat agree 44.8% (95% CI 42.5, 47.1), Neither 21.6% (95% CI 19.7, 23.6), Somewhat disagree 7.9% (95% CI 6.7, 9.3) and Strongly disagree 2.6% (95% CI 1.9, 3.5).

### Types of experiences in different domains

Table 2 gives the proportions of respondents reporting experiences in specific domains. While some of the questions varied according to domain, the most common experience reported by participants was that people in the following domains did not understand the impact of their mental health problem: partners (56.0% [95% CI 51.9, 60.1]), friends (52.5% [95% CI 49.2, 55.8]), family (60.8% [95% CI 57.2, 64.3]) and the workplace (42.7% [95% CI 38.7, 46.8]). Other common experiences in the partners and family domains related to people being judgemental and getting angry, while friends were more likely to be reported as avoiding or cutting contact with the person (42.3% [39.1, 45.5]).

**Table 2.** Discrimination experiences in specific domains

Discrimination experiences in different domains	% [95% CI]
**Partner/husband/wife (*n* = 840)**	
They did not understand the impact of my mental health problem	56.0 [51.9, 60.1]
They were judgemental	43.5 [39.5, 47.7]
They got angry at me	42.2 [38.2, 46.3]
They were insulting towards me	29.4 [25.8, 33.2]
They assumed that any of my ‘bad days’ were related to my mental health problem	22.1 [18.9, 25.7]
They avoided or cut contact with me	21.5 [18.3, 25.1]
They did not believe I had a mental health problem	19.6 [16.4, 23.1]
**Friends (*n* = 1329)**	
They did not understand the impact of my mental health problem	52.5 [49.2, 55.8]
They avoided or cut contact with me	42.3 [39.1, 45.5]
They were judgemental	31.6 [28.6, 34.7]
They assumed that any of my ‘bad days’ were related to my mental health problem	18.2 [15.8, 20.8]
They did not believe I had a mental health problem	16.1 [13.8, 18.6]
They got angry at me	12.6 [10.5, 15.1]
They were insulting towards me	11.9 [9.8, 14.3]
**Family (*n* = 1074)**	
They did not understand the impact of my mental health problem	60.8 [57.2, 64.3]
They were judgemental	46.9 [43.3, 50.6]
They got angry at me	31.9 [28.5, 35.5]
They did not believe I had a mental health problem	29.1 [25.8, 32.5]
They were insulting towards me	27.4 [24.2, 30.9]
They avoided or cut contact with me	23.0 [20.2, 26.1]
They assumed that any of my ‘bad days’ were related to my mental health problem	19.1 [16.5, 22.1]
**Social media (*n* = 471)**	
They were judgemental	54.9 [49.5, 60.2]
They were insulting towards me	37.0 [31.9, 42.4]
They did not understand the impact of my mental health problem	27.3 [22.7, 32.4]
They did not believe I had a mental health problem	17.2 [13.4, 21.8]
They avoided or cut contact with me	14.8 [11.6, 18.7]
They got angry at me	13.4 [10.0, 17.6]
They denied me access to groups/online communities	11.2 [8.3, 14.9]
They assumed that any of my ‘bad days’ were related to my mental health problem	7.3 [5.0, 10.6]
**Workplaces (*n* = 833)**	
My employer or colleagues did not understand the impact of my mental health problem	42.7 [38.7, 46.8]
I was denied opportunities	36.3 [32.4, 40.4]
My employer or colleagues weren’t supportive of my needs	32.7 [29.0, 36.6]
My employer or colleagues treated me as if I was incompetent	27.5 [24.1, 31.3]
My employer or colleagues avoided or excluded me	20.7 [17.7, 24.1]
I was forced to change responsibilities	20.0 [16.8, 23.7]
I was fired or made redundant	14.0 [11.5, 17.0]
My employer or colleagues did not believe I had a mental health problem	12.5 [9.9, 15.5]
**Health professionals (*n* = 766)**	
They were dismissive or sceptical	61.0 [56.7, 65.2]
They lacked an understanding of my mental health problem	38.2 [34.0, 42.6]
They were judgemental	36.6 [32.5, 40.9]
They were not willing to listen	35.0 [31.0, 39.3]
They were not supportive or caring	34.2 [30.2, 38.5]
They ignored my physical health problems	31.8 [28.0, 35.9]
There was a delay or problem in helping me access care	24.0 [20.4, 28.0]
They prescribed medication without adequate explanation information consultation	21.2 [17.9, 25.1]
They tried to avoid dealing with me	15.5 [12.4, 19.1]
They refused to prescribe medication	11.6 [9.1, 14.5]

In workplaces, being denied opportunities (36.3% [95% CI 32.4, 40.4]) and having employers or colleagues that were not supportive of the person’s needs (32.7% [95% CI 29.0, 36.6]) were also relatively common. People on social media were most commonly reported as being judgemental (54.9% [95% CI 49.5, 60.2] or insulting (37.0% [95% CI 31.9, 42.4]).

Health professionals were most commonly reported as being dismissive or sceptical (61.0% [95% CI 56.7, 65.2]). When asked about the type of health professional from which the person experienced discrimination, by far the most common were GPs (69.5% [95% CI 65.3, 73.4]). Other proportions were as follows: psychiatrists (13.9% [95% CI 11.2, 17.3]), psychologists (13.3% [95% CI 10.6, 16.7]), emergency department doctors (12.7% [95% CI 10.0, 16.0]), nurses (11.9% [95% CI 9.4, 15.1]) and counsellors/psychotherapists (11.3% [95% CI 8.8, 14.3]).

### Sociodemographic and mental health problem characteristics associated with the burden of discrimination in different domains

Table 3 gives the sociodemographic and mental health problems characteristics associated with the burden score in different domains. Compared to those aged 18–34 years, those aged 35–64 years were more likely to experience a higher burden in social life, dating/intimate partners, family, finding a job, keeping a job, health professionals (both mental and physical problems) and the legal system. Those aged 65+ had a higher burden in finding and keeping a job. Females experienced a lower burden in relation to the legal system. Those with a higher level of education experienced a higher burden in relation to keeping a job and a lower burden in relation to housing. Those from non-English speaking backgrounds experienced a lower burden in relation to starting a family, education or training and welfare benefits. Those with a self-reported anxiety problem experienced a higher burden in relation to making or keeping friends and social life; those with a severe mental illness experienced a higher burden in finding or keeping a job and those with depression experienced a higher burden in making or keeping friends, social life, dating/partner, health professionals (physical health problems) and the legal system. Those with ADHD experienced a higher burden in social life, family, health professionals (mental and physical health problems), welfare benefits and the legal system.

**Table 3.** Sociodemographic and mental health condition factors associated with the burden of discrimination in different domains

	Making or keeping friends *B* (95% CI)	Social life *B* (95% CI)	Dating or partner/ husband/wife *B* (95% CI)	Starting a family or having children *B* (95% CI)	Family *B* (95% CI)	People in your neighbourhood *B* (95% CI)	Finding a job *B* (95% CI)	Keeping a job *B* (95% CI)	Education or further training *B* (95% CI)	Health professional (mental health problem) *B* (95% CI)	Health professional (physical health problems) *B* (95% CI)	Housing (incl. renting, public housing) *B* (95% CI)	Welfare benefits or disability pensions *B* (95% CI)	Legal system *B* (95% CI)	Social media *B* (95% CI)
**Age group (reference age 18–34)**															
35−64 years	0.29 [−0.02, 0.60]	0.34 [0.08, 0.60]*	0.45 [0.04, 0.87]*	0.66 [−0.06, 1.37]	0.33 [0.03, 0.63]*	0.45 [−0.05, 0.95]	0.64 [0.16, 1.12]*	1.00 [0.55, 1.45]***	0.48 [−0.01, 0.97]	0.68 [0.16, 1.19]*	0.79 [0.43, 1.16]***	0.46 [−0.15, 1.08]	0.55 [−0.05, 1.15]	1.12 [0.55, 1.69]***	0.32 [−0.09, 0.72]
65+	−0.26 [−0.70, 0.19]	0.10 [−0.27, 0.47]	−0.14 [−0.69, 0.42]	−0.37 [−2.11, 1.36]	−0.12 [−0.54, 0.30]	0.36 [−0.36, 1.08]	2.10 [0.74, 3.46]**	1.78 [0.70, 2.85]**	0.21 [−0.68, 1.09]	0.30 [−0.55, 1.15]	0.06 [−0.49, 0.61]	0.22 [−1.24, 1.68]	−0.08 [−0.85, 0.70]	−0.01 [−0.79, 0.76]	−0.32 [−0.83, 0.19]
Gender (reference male)															
Female	−0.13 [−0.43, 0.17]	−0.04 [−0.29, 0.22]	0.05 [−0.34, 0.44]	−0.18 [−0.94, 0.57]	0.14 [−0.13, 0.41]	−0.08 [−0.54, 0.38]	−0.44 [−0.93, 0.05]	−0.26 [−0.71, 0.18]	−0.24 [−0.78, 0.30]	−0.32 [−0.85, 0.20]	0.29 [−0.11, 0.69]	−0.28 [−0.98, 0.43]	−0.13 [−0.66, 0.40]	−0.59 [−1.16, −0.03]*	−0.08 [−0.48, 0.32]
Prefer another term	0.57 [−0.40, 1.54]	0.20 [−0.63, 1.04]	0.82 [−0.76, 2.40]	1.79 [−2.26, 5.83]	0.40 [−0.64, 1.44]	−0.01 [−1.11, 1.09]	−0.26 [−1.30, 0.78]	0.49 [−0.81, 1.79]	0.10 [−0.97, 1.16]	−0.01 [−0.90, 0.88]	0.40 [−0.31, 1.10]	−0.34 [−1.62, 0.94]	0.17 [−1.14, 1.49]	0.35 [−0.73, 1.42]	−0.03 [−1.17, 1.11]
Education (reference below bachelor)															
Bachelor or above	0.05 [−0.22, 0.32]	−0.02 [−0.24, 0.19]	−0.21 [−0.56, 0.13]	−0.13 [−0.84, 0.58]	−0.28 [−0.58, 0.01]	−0.25 [−0.64, 0.14]	0.29 [−0.21, 0.78]	0.49 [0.01, 0.96]*	0.07 [−0.36, 0.50]	−0.20 [−0.77, 0.37]	0.09 [−0.24, 0.41]	−0.73 [−1.40, −0.07]*	−0.12 [−0.73, 0.48]	0.20 [−0.32, 0.72]	−0.04 [−0.41, 0.33]
Country of birth (reference main English speaking)															
NESB	−0.18 [−0.60, 0.23]	0.07 [−0.30, 0.44]	−0.33 [−0.91, 0.24]	−1.30 [−2.49, −0.11]*	−0.23 [−0.64, 0.17]	−0.04 [−0.58, 0.49]	−0.62 [−1.40, 0.15]	−0.55 [−1.31, 0.21]	−0.63 [−1.22, −0.05]*	−0.18 [−0.87, 0.52]	−0.23 [−0.76, 0.29]	−0.34 [−1.14, 0.47]	−0.86 [−1.48, −0.24]**	−0.24 [−0.98, 0.49]	−0.37 [−0.79, 0.05]
Aboriginal and Torres Strait Islander (reference yes)															
No	−0.50 [−1.08, 0.09]	−0.38 [−0.91, 0.14]	0.26 [−0.69, 1.21]	0.25 [−1.22, 1.71]	0.08 [−0.58, 0.73]	0.21 [−0.48, 0.90]	−0.48 [−1.31, 0.35]	−0.39 [−1.13, 0.36]	−0.80 [−2.08, 0.49]	−0.23 [−1.10, 0.64]	−0.26 [−0.94, 0.41]	0.11 [−0.90, 1.13]	−0.58 [−1.44, 0.28]	0.05 [−1.02, 1.12]	−0.64 [−1.60, 0.32]
Mental health condition^a^															
Any anxiety condition	0.36 [0.02, 0.70]*	0.44 [0.21, 0.67]***	0.36 [−0.05, 0.77]	0.19 [−0.98, 1.37]	0.30 [−0.04, 0.63]	0.13 [−0.59, 0.85]	0.00 [−0.70, 0.70]	0.13 [−0.42, 0.69]	0.26 [−0.57, 1.09]	−0.36 [−1.11, 0.39]	−0.42 [−1.10, 0.26]	−0.27 [−1.13, 0.59]	0.91 [−0.02, 1.84]	0.38 [−0.48, 1.23]	0.09 [−0.51, 0.69]
Any severe mental health condition	0.28 [−0.16, 0.72]	0.26 [−0.11, 0.63]	0.50 [−0.23, 1.23]	0.49 [−0.80, 1.78]	0.24 [−0.19, 0.67]	0.45 [−0.29, 1.19]	0.66 [0.01, 1.31]*	0.77 [0.22, 1.31]**	0.09 [−0.81, 1.00]	0.50 [−0.13, 1.12]	0.31 [−0.14, 0.77]	0.70 [−0.29, 1.69]	0.05 [−0.65, 0.74]	0.52 [−0.39, 1.44]	0.45 [−0.24, 1.14]
Depression	0.29 [0.03, 0.55]*	0.28 [0.06, 0.50]*	0.47 [0.10, 0.84]*	0.02 [−0.73, 0.76]	0.20 [−0.09, 0.49]	0.22 [−0.24, 0.68]	0.27 [−0.18, 0.72]	0.51 [0.09, 0.93]*	0.33 [−0.19, 0.84]	0.07 [−0.44, 0.57]	0.44 [0.10, 0.78]*	−0.23 [−0.97, 0.51]	0.16 [−0.45, 0.76]	0.60 [0.02, 1.17]*	−0.19 [−0.58, 0.21]
ADHD	0.32 [−0.04, 0.68]	0.52 [0.21, 0.83]**	0.32 [−0.14, 0.78]	0.00 [−0.70, 0.69]	0.58 [0.21, 0.94]**	0.37 [−0.23, 0.97]	0.54 [−0.00, 1.08]	0.34 [−0.17, 0.85]	0.45 [−0.07, 0.96]	0.83 [0.24, 1.42]**	0.39 [0.00, 0.77]*	0.64 [−0.03, 1.32]	0.75 [0.13, 1.36]*	0.87 [0.23, 1.51]**	0.00 [−0.41, 0.41]

a Mental health problems coded as present vs absent.

* *p* < 0.05, **p < 0.01, ***p < 0.001 (in bold type). Regression coefficients are unstandardized.

## Discussion

To our knowledge, this is the first nationally representative survey to explore the frequency and impact of discrimination related to mental health problems in a wide range of life domains. While discrimination in the domains of social life and family was most common, the burden of discrimination, as a product of the frequency and severity of impact, was greatest in the domains of finding and keeping a job, in dating/intimate relationships, in housing (including renting or public housing) and in welfare benefits or disability pensions. The most common type of discrimination experience in the workplace and among friends, family and partners was of having people lack understanding of the impact of their mental health problem. Overall, 67.7% (95% CI 65.5, 69.9) agreed that stigma and discrimination was worse than the mental health problem itself.

Findings from this study are in line with previous population-level surveys that show that discrimination in social life, family and making or keeping friends are the most frequent experiences, with population proportions in this study of 43.6%, 41.5% and 41.0% respectively (Stuart *et al.*, 2014; Wong *et al.*, 2017; Lasalvia *et al.*, 2021). It also extends these by highlighting the impact of discrimination in relation to dating or intimate relationships, which, in this study, had the second highest burden and was more common in people aged 35–64 years and those with a diagnosis of depression. There is some evidence that mental health problems are seen as dating and relationship ‘dealbreakers’ (Boysen *et al.*, 2019). Despite this, relatively little attention has been paid to supporting people with mental health problems, particularly those that are more severe, to find and sustain intimate relationships (Henderson *et al.*, 2026). In cases where a person is in a long-term intimate relationship and develops a mental health problem, relationship breakdown may be more likely, and this may also underlie the high burden seen in this study (Kessler *et al.*, 1998). The finding that the most common experience in dating and intimate relationships was of partners lacking understanding about the impact of the person’s mental health problems points to the need for interventions to support people caring for a person with a mental health problem, as well as for people with mental health problems to both develop and maintain intimate relationships (Henderson *et al.*, 2026).

This study also reinforces and extends others that point to the importance of reducing discrimination and improving inclusion of people with mental health problems in workplaces, particularly in those with depression and more severe mental health problems, as these were associated with a higher burden in the current study. For example, in a European multi-country study, over 20% of people with a diagnosis of major depressive disorder reported discrimination in the area of keeping a job (Lasalvia *et al.*, 2013). Reducing discrimination in workplaces is likely to require attention to structural discrimination – workplace policies and practices that promote exclusion – as well as interventions to reduce discriminatory interpersonal interactions (Nogues and Finucan, 2018). The former can build on and strengthen legislation to ensure reasonable adjustments for people who disclose mental health problems, as well as transparent, streamlined processes for managing complaints. Initiatives to ensure that employers and employees are aware of their rights and responsibilities in relation to health, safety and anti-discrimination policies are also essential (Thornicroft *et al.*, 2022). While many workplaces may be implementing these fairly and effectively, the proportion of people in the current survey reporting these experiences and the impact of them point to the need for improvements. Recognizing the need, many organizations provide training for managers on how to reduce work-based mental health risks for employees. While there is evidence that these can improve stigmatizing attitudes, there has been relatively little exploration of the impacts on experiences of discrimination and support in employees themselves (Gayed *et al.*, 2018). Further research using rigorous designs that involve the collection of data from managers, employees and operational data where relevant and appropriate are needed. As an example, a cluster randomized trial of a manager mental health training program in a large Australian fire and rescue service showed that the employees whose managers were in the intervention arm had reduced sick leave compared to those in the control arm, potentially because they were better supported by managers (*p* = 0.049) (Milligan-Saville *et al.*, 2017). Future research could use similar study designs to explore the effectiveness and cost-effectiveness of programs that include evidence-based anti-stigma elements and that explore experiences of discrimination and support in employees as primary outcomes.

The finding that the most common type of discrimination experience for people in the workplace was of having people lack understanding of their mental health problem also aligns with other studies (Brouwers *et al.*, 2016; Van Bortel *et al.*, 2024). It points to the need for interventions to support managers to feel comfortable to have conversations that explore the specific impacts of mental health problems at an individual level, given that these are likely to vary widely. These can be challenging for many managers who may fear offending the person, making the situation worse or encountering legal problems. Training programs such as Mental Health First Aid (MHFA) have a valuable role to play in overcoming such challenges and have been widely implemented in workplaces. MHFA training aims to support the general public to help a person who may be experiencing a mental health problem or in a crisis in a non-stigmatizing and supportive way and is an effective anti-stigma intervention (Morgan *et al.*, 2018). Employees with mental health problems may also require support to disclose or have discussions with their employers (Hastuti and Timming, 2021). Further research is also needed to improve structures and processes that support people with mental health problems as they seek employment. This is likely to include support for individuals, particularly those with complex mental illness, as well as hiring managers and HR staff (Henderson *et al.*, 2026).

A similarly high discrimination burden was also reported in the domain of housing, with a higher burden seen in those with lower (below bachelor) levels of education. This aligns with evidence from a Canadian study showing that among people with mental health problems, discrimination was related to housing stability (Mejia-Lancheros *et al.*, 2021). While this relationship may be stronger among people with more severe mental illness, who are often at greater risk of homelessness (Morgan *et al.*, 2012), over 15% of respondents reported discrimination in this domain in the current survey (in which most respondents reporting common mental health problems). This suggests that people with these problems are also impacted, and further research is needed to explore these experiences and how to address them, particularly in a housing shortage such as that occurring in most parts of Australia.

Discrimination in relation to welfare benefits or disability pensions also had a high burden for respondents. Having a mental health problem increases the risk of poverty, and access to state benefits is often essential for recovery. Studies in a number of countries have explored experiences of discrimination in this domain, although these have typically been surveys of service users or qualitative studies (Hamilton *et al.*, 2016; Galloway *et al.*, 2018). While the current study did not have a deeper exploration of the types of experiences in this domain, they align with reports of poor treatment by employees in welfare agencies such as Centrelink and also in those accessing or attempting to access support from Australia’s National Disability Insurance Scheme (NDIS) (Isaacs *et al.*, 2024). In the current study, those reporting a diagnosis of ADHD reported higher levels of discrimination in relation to welfare benefits, which may reflect challenges accessing the NDIS in this population (Isaacs *et al.*, 2024). Interestingly, those from non-English speaking backgrounds reported less discrimination from welfare providers, potentially because they may have come from countries with limited welfare provisions. Attention to structural and interpersonal reforms that include a greater focus on tackling challenges in application processes, person-centred care and engagement with consumers and carers, as well as training for workers in these organizations, is likely to be essential to reducing discrimination in this domain (Elmes *et al.*, 2021; Mellifont *et al.*, 2022). These may include working groups, shared accountability frameworks, cross-sector policy mechanisms and the development of joint indicators of success as well as mechanisms for ongoing monitoring and public reporting (Thornicroft *et al.*, 2022).

The study has several strengths. It is the first to assess, at a population level, the burden of actual experiences of discrimination in key life domains. However, burden scores were only calculated for the subset of the sample that had relevant experience to report (i.e., for a person not in employment or not in the rental market/public housing no score could be calculated). Thus, the burden scores cannot be applied evenly in the population. For some population groups (notably those with severe mental health conditions), the sample size was relatively small, leading to greater uncertainty in estimates. Additionally, the higher burden seen for people reporting some diagnoses potentially considered less ‘severe’ in some settings (e.g., making or keeping friends, social life or dating) may reflect the fact that people with these conditions have more opportunities for experiences. It is also possible that people with more severe conditions ‘expect’ to have negative experiences and do not therefore characterize them as discriminatory, potentially due to self-stigma (Dubreucq *et al.*, 2021). Moreover, illness categories were coded as present vs absent, and we did not collect information on functional impairment. A further strength is that, as the survey collected data from the general population, it is also less likely to be biased towards over-reporting due to people who have experienced discrimination being more likely to volunteer to take part in a survey on the topic. However, the proportion of people meeting the criteria for high distress or an in-scope diagnosis was, at 44%, higher than the prevalence data from Australia’s National Study of Mental Health and Wellbeing would have suggested (Slade *et al.*, 2025). This may reflect ‘diagnostic expansion’ (where people give diagnostic labels to their experiences even though they may not meet criteria for a formal diagnosis (Reavley *et al.*, 2025), the inclusion of diagnoses (e.g., ADHD) not included in the National Study or people with symptoms but no reported diagnosis. It is also possible that this diagnostic expansion may result in increased sensitivity to harm in the form of discrimination (Haslam, 2016; Foulkes and Andrews, 2023). For the stigma field, these findings raise important questions about recognizing legitimate harm without overextending stigma-related concepts. This issue requires further research, and approaches to stigma reduction should carefully consider both benefits and potential harms (Reavley, 2026).

However, generalizability is limited by likely lower levels of participation from those with more severe mental health conditions or symptoms or lower digital access. Other limitations include the lack of further detail on experiences in particular domains, including welfare benefits and housing.

## Conclusions

More than two-thirds of people in the current study agreed that stigma and discrimination was worse than the impact of their mental health problem. Findings suggest that reducing the frequency and impact of discrimination in workplaces, welfare benefits and housing should be key targets for policy and practice, including in Australia’s National Stigma and Discrimination Reduction Strategy. In workplaces, the focus should be on ensuring that employers and employees are aware of their rights and responsibilities in relation to health, safety and anti-discrimination policies. Transparent, streamlined processes for managing complaints are also likely to be critical. Similarly, policies in welfare, social services and housing settings should also be reviewed to ensure that they are operating consistently with the social model of disability and provide person-centred and culturally safe services. Employees in workplaces, particularly those in relevant agencies, should also be provided with evidence-based, lived experience-informed, anti-stigma training. Where possible, cross-sector policy mechanisms should be considered to strengthen such approaches (Thornicroft *et al.*, 2022). Improving the capacity of intimate partners, families and friendship groups to better understand the impacts of mental health problems on individuals, which may be best tackled through population-level campaigns, should also be a priority.

## Supporting information

10.1017/S2045796026100456.sm001 Reavley et al. supplementary material Reavley et al. supplementary material

## Data Availability

Data are available on reasonable request.

## Supplementary material

The supplementary material for this article can be found at https://doi.org/10.1017/S2045796026100456.
