# Supplementary material for: A nationally representative survey of the impact of discrimination towards people with mental health problems: SANE’s 2025 National Stigma Report Card
Source: Epidemiol Psychiatr Sci. 2026 Feb 3;35:e9. doi: 10.1017/S2045796026100456 (PMC12925686; doi:10.1017/S2045796026100456)
Supplement: Reavley et al. supplementary material [file S2045796026100456sup001.docx]

## Supplementary material: discrimination burden score

To create a ‘discrimination burden score’ for each domain, frequency of experiences of discrimination (scored 1= a little, 2=moderately and 3=a lot respectively) was multiplied by the severity of impact (with the response options scored as follows: -2=large positive impact, -1=small positive impact, 0= no impact, 1=small negative impact, 2= large negative impact. Scores ranged from -6 to +6. Higher scores indicated a higher burden.

As an example, we provide a table with the spread of scale scores with 95% CIs for the friends domain.

Figure S1 Spread of burden scale scores in the friends domain
